# Molecular Alterations and Their Correlation With the Survival of Glioblastoma Patients With Corpus Callosum Involvement

**DOI:** 10.3389/fnins.2021.701426

**Published:** 2021-07-30

**Authors:** Meng Cui, Xin Gao, Yihong Chi, Meng Zhang, Hepu Lin, Hewen Chen, Caihong Sun, Xiaodong Ma

**Affiliations:** ^1^Medical School of Chinese PLA, Beijing, China; ^2^Department of Neurosurgery, The First Medical Centre, Chinese PLA General Hospital, Beijing, China; ^3^Department of Information Technology, Xian Janssen Pharmaceutical Ltd., Beijing, China; ^4^Department of Neurosurgery, The Seventh Medical Center of PLA General Hospital, Beijing, China

**Keywords:** glioblastoma, corpus callosum, *PDGFRA* amplification, *PDGFRA* mutation, survival

## Abstract

**Purpose:** To explore molecular alterations and their correlation with the survival of patients with glioblastoma (GBM) with corpus callosum (CC) involvement (ccGBM).

**Methods:** Electronic medical records were reviewed for glioma patients tested for molecular alterations and treated at our hospital between January 2016 and July 2020. ccGBM was compared to GBM without CC involvement (non-ccGBM) to identify differences in molecular alterations. Clinical outcomes and survival were compared between ccGBM and non-ccGBM patients, as well as among patients with ccGBM with different molecular alteration statuses. ccGBM was also compared to diffuse midline glioma (DMG) to clarify their correlation in molecular alterations, the progression-free survival (PFS), and overall survival (OS).

**Results:** Thirty ccGBM and 88 non-ccGBM patients were included. *PDGFRA* amplification (*PDGFRAamp*, 33.3 vs. 9.1%, *P* = 0.004) and missense mutation (*PDGFRAmut*, 20.0 vs. 3.4%, *P* = 0.011) both had higher incidences in ccGBM than in non-ccGBM. *PDGFRA* alteration was associated with the occurrence of ccGBM (OR = 4.91 [95% CI: 1.55–15.52], *P* = 0.007). ccGBM with *PDGFRAamp* resulted in a shorter median PFS (8.6 vs. 13.5 months, *P* = 0.025) and OS (12.4 vs. 17.9 months, *P* = 0.022) than non-ccGBM with *PDGFRAnon-amp*. ccGBM with *PDGFRAamp* combined with *PDGFRAmut* (*PDGFRAamp-mut*) had a shorter median PFS (7.6 vs. 8.9 months, *P* = 0.022) and OS (9.6 vs. 17.8 months, *P* = 0.006) than non-ccGBM with wild-type *PDGFRA* and no amplification (*PDGFRA-w, non-amp*). Compared to ccGBM with *PDGFRA-w, non-amp*, ccGBM with *PDGFRAamp* and *PDGFRAamp-mut* both had a shorter median PFS and OS (*P* < 0.05). The hazard ratios (HRs) of *PDGFRAamp* for PFS and OS in ccGBM were 3.08 (95% CI: 1.02–9.35, *P* = 0.047) and 5.07 (1.52–16.89, *P* = 0.008), respectively, and the HRs of *PDGFRAamp-mut* for PFS and OS were 13.16 (95% CI: 3.19–54.40, *P* < 0.001) and 16.36 (2.66–100.70, *P* = 0.003). ccGBM may have similar incidences of *PDGFRAamp* or *mut* (*PDGFRAamp/mut*) as DMG, and they also had similar median PFS (10.9 vs. 9.0 months, *P* = 0.558) and OS (16.8 vs. 11.5 months, *P* = 0.510).

**Conclusion:***PDGFRA* alterations are significantly associated with the occurrence and poor prognosis of ccGBM. ccGBM with *PDGFRAamp/mut* may be classified as a single subtype of GBM that has a similar survival rate to DMG. PDGFR inhibitors may be a promising treatment method for ccGBM.

## Introduction

Glioblastoma (GBM) is highly malignant tumor (World Health Organization [WHO] grade IV), with an annual incidence of 3.1 per 100,000 and a 5-year survival rate of less than 3% (Ohgaki, 2009; Wirsching et al., 2016). GBM is very invasive, typically infiltrating along white matter tracts (Giese and Westphal, 1996; Giese et al., 2003). As the largest interhemispheric fiber bundle in the human brain, the corpus callosum (CC) is frequently invaded by GBM (Nazem-Zadeh et al., 2012). GBM with CC involvement (ccGBM) can be classified into two types. In one type, the lesion invades only one side of the hemisphere and the CC (non-butterfly ccGBM). The other type is known as butterfly GBM (bGBM) and involves the corpus callosum and both cerebral hemispheres (Louis et al., 2007; Nazem-Zadeh et al., 2012; Ho et al., 2013; Komori, 2017). ccGBM has a poorer prognosis than GBM without CC involvement (non-ccGBM) and is associated with incomplete resection or residual tumor after surgery (Talos et al., 2006). Whether the overall survival (OS) rate of ccGBM can be prolonged by surgical resection has been controversial (Dziurzynski et al., 2012; Chaichana et al., 2014; Dayani et al., 2018; Opoku-Darko et al., 2018; Franco et al., 2020). The present consensus is that ccGBM has a poorer prognosis than common GBM regardless of whether resection is performed (Chen et al., 2015; Liang et al., 2016).

At present, the WHO classification of glioma is based on pathological diagnosis and analysis of molecular alterations, which provides a more precise diagnosis and better comprehension of the type of glioma. Previous studies have revealed the association between some molecular alterations and the survival rate of glioma. For example, glioma with *IDH* or *TERT* mutation has a better prognosis, and glioma with *H3F3A* or *HIST1H3B/C* mutation is classified as diffuse midline glioma and has a poorer prognosis (Onizuka et al., 2020). While the molecular characteristics of non-ccGBM are established, the molecular characteristics of ccGBM were unknown until now. In this study, we aimed to explore the molecular alterations in ccGBM and determine their association with survival.

## Materials and Methods

### Patient Selection

Retrospective clinical data of 1232 glioma patients were obtained from electronic medical records (EMRs) in the Department of Neurosurgery at our hospital between January 2016 and July 2020. The enrollment criteria were patients 18–80 years of age who underwent resection at our hospital, who were diagnosed with supratentorial WHO II-IV grade gliomas by pathology, received testing of the molecular alteration status of the tumor, had extent of resection (EOR) values calculated and attended postoperative follow-up regularly. The exclusion criteria were as follows: (1) patients with infratentorial glioma, (2) patients who underwent only biopsy, (3) patients for whom the pathological diagnosis could not be confirmed, (4) patients with diffuse midline glioma, and (5) patients lost to follow-up. This study was carried out according to the principles of the Helsinki Declaration and approved by our institutional ethics committee (No. S2014-096-01). Written informed consent for surgery and molecular testing was previously provided by all patients or their relatives.

### Patient Groupings

The included patients were divided into the glioma invading the corpus callosum (ccGlioma) group and the non-ccGlioma control group. The incidences of molecular alterations between the two groups were compared to identify the molecular differences and the significant molecular alterations in ccGBM. Then, the patients with WHO grade IV GBM were divided into the ccGBM group and non-ccGBM group. To explore the association between significant molecular alterations and the survival rate of GBM, these two groups were matched according to age, sex, *IDH* mutation (*IDHmut*) status, *TERT* mutation (*TERTmut*) status, *MGMT* promoter methylation (*MGMTmet*) status, EOR value, radiotherapy, and cycles of temozolomide (TMZ) chemotherapy. The ccGBM group was also divided into different groups according to significant molecular alterations to identify the effect of different molecular characteristics on the survival rate. ccGBM was also compared to diffuse midline glioma (DMG) to clarify the correlation between molecular alterations and survival.

### Volumetric Analysis

MRI was performed for all patients using a 1.5 Tesla (T) scanner (Siemens Espree, Erlangen, Germany). The digital imaging and communications in medicine (DICOM) data of all MRI sequences were transferred to iPlan software 2.6 (Brainlab Feldkirchen, Germany). Pre-and postoperative tumor volumes (cm3) were calculated automatically by the software based on the tumor tissue seen on postcontrast 3D T1-weighted MR images of GBM. The EOR was defined as follows: (preoperative tumor volume – postoperative residual tumor volume)/preoperative tumor volume × 100.

### Next-Generation DNA Sequencing (NGS)

Tumor samples were examined by neuropathologists to confirm the histopathological diagnosis and WHO grade based on the 2016 WHO classification system (Louis et al., 2016). Genomic DNA (gDNA) was extracted from fresh tumor tissues using a QIAamp DNA Mini Kit (Qiagen 51306, Germany) and quantified by UV absorption (Nanodrop, Thermo Scientific, Wilmington, DE, United States). Sixty-eight gene mutation statuses were tested in this study (Genetron Health; Beijing, China). Libraries of gDNA were enriched for the tested regions using a custom-designed capture probe. Prepared libraries (750 ng) were hybridized with two different hybridization reagents in blocking agents using the SureSelectXT Target Enrichment System (Agilent Technologies). The enriched libraries were amplified with the P5/P7 primer. After performing a quality control analysis with a 2200 Bioanalyzer and quantification with a Qbit3 and a qPCR NGS library quantification kit (Agilent Technologies), the libraries were sequenced on a HiSeq X10 platform (Illumina, San Diego, CA, United States).

Primary processing of NGS data for tissue samples was performed using trimmomatic methods (0.36), including demultiplexing and masking of dual-index adapter sequences. Sequence reads were aligned against the human reference genome (version GRCh37/hg19) using BWA (version 0.7.10). Candidate somatic mutations consisting of point mutations, small insertions, and deletions were identified by SAMtools (version 1.3.1) and pindel (version 0.2.5b8, 20151210) across the targeted regions of interest. Integrative Genomics Viewer (IGV) was applied to filter alignment and sequencing artifacts.

### Postoperative Treatment and Follow-Up

Radiotherapy plus concomitant and adjuvant TMZ chemotherapy for GBM were recommended (Stupp et al., 2005; Duffau and Taillandier, 2015). Regular MRI scans and follow-up were performed every 3 months. The end point of follow-up was March 2021. The progression-free survival and overall survival rates were calculated according to the results of the follow-up.

### Statistical Analysis

Statistical analysis was performed using SPSS 21.0 software (SPSS Inc., Chicago, IL, United States). The Shapiro-Wilk test was used to test the normality of the data. Student’s *t* and χ^2^ (or Fisher’s exact test) tests were used to compare continuous parametric and categorical variables between groups, respectively. The Mann-Whitney *U*-test was used to compare continuous non-parametric variables, such as the EOR value, between groups. Univariate and multivariate logistic regression were used to evaluate the correlation between ccGBM and clinical factors and molecular alterations. Survival curves were estimated by the Kaplan–Meier method and were compared by the log-rank test. Cox proportional hazard models were used to identify significant prognostic factors for PFS and OS. A *P* value < 0.05 was considered statistically significant.

## Results

### Molecular Alterations of ccGlioma and Non-ccGlioma

**FIGURE 1:**
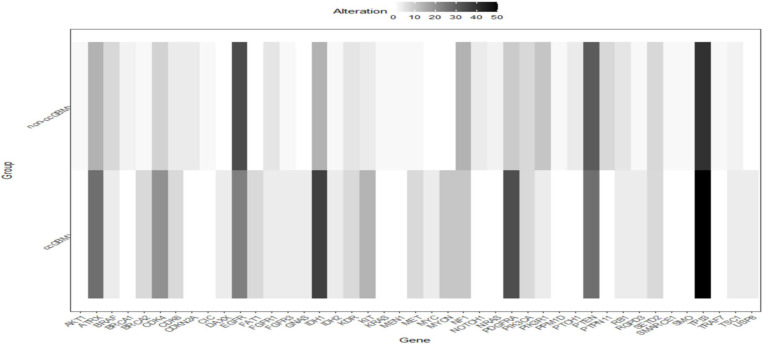
Heatmap of incidences of molecular alterations between ccGBM and non-ccGBM. The horizontal axis represented different genes, the longitudinal axis represented the two groups of ccGBM and non-ccGBM. Low incidences of alterations were shown in white, high incidences in black.

**FIGURE 2:**
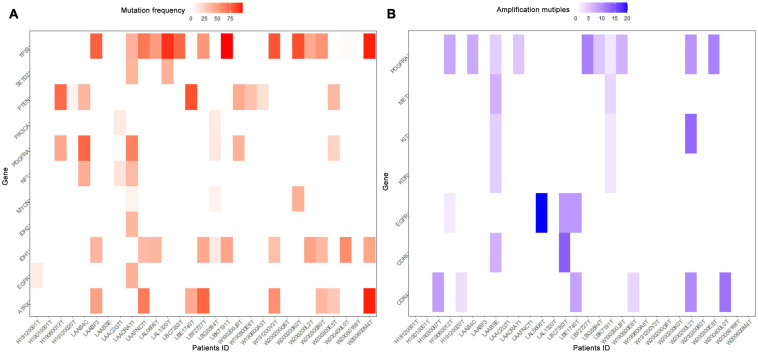
Heatmap of molecular mutations frequencies **(A)** and amplification multiples **(B)** in ccGBM. The horizontal axis represented the identification number of tumor samples, the longitudinal axis represented related genes. Low frequencies and multiples were shown in white, high frequencies and multiples in red and blue, respectively.

A total of 223 patients who underwent molecular alteration testing were finally included, among whom 47 had gliomas with CC involvement. The incidences of molecular alterations are presented in Table 1, in which the ccGlioma group and the non-ccGlioma group are compared. The mutation incidences of *BCOR*, *PPM1D*, and *TERT* in the patients in the ccGlioma group with WHO grade III tumors were higher than those in the non-ccGlioma group. The mutation incidence of *IDH 1* or *2* in the ccGBM group was higher than that in the non-ccGBM group. The alteration incidences of *MYCN* and *PDGFRA* in the ccGBM group were higher than those in the non-ccGBM. The ccGBM group had a higher incidence of *PDGFRA* amplification (*PDGFRAamp*) (33.3 vs. 9.1%, *P* = 0.004) and *PDGFRA* mutation (*PDGFRAmut*) (20.0 vs. 3.4%) than the non-ccGBM group. Among 10 ccGBM patients with *PDGFRA* alterations, 10 patients had *PDGFRAamp*, and 6 patients had 7 missense mutations. All ccGBM patients with *PDGFRAmut* had *PDGFRAamp*. Other molecular alterations that were significantly associated with the prognosis of glioma are also shown in Table 1, but none of them were identified to be different between the ccGlioma group and non-ccGlioma group (Figure 1). The multivariate analysis showed that only the *PDGFRA* alteration was associated with the occurrence of ccGBM (odds ratio [OR]: 4.91 [1.55–15.52], *P* = 0.007) (Table 2). The detailed results of molecular alterations in ccGBM are shown in Figure 2.

**TABLE 1:** Molecular alterations of ccGlioma and non-ccGlioma of different WHO grades by univariate logistic regression.

**Molecular alterations (N [%])**	**ccGlioma (WHO II) *N* = 6**	**Non-ccGlioma (WHO II) *N* = 42**	***P* value**	**ccGlioma (WHO III) *N* = 11**	**Non-ccGlioma (WHO III) *N* = 46**	***P* value**	**ccGBM (WHO IV) *N* = 30**	**Non-ccGBM (WHO IV) *N* = 88**	***P* value**
*BCORmut*	0 (0)	0 (0)	/	3 (27.3)	1 (2.2)	**0.020**	0 (0)	0 (0)	/
*MYCN alteration*	0 (0)	0 (0)	/	0 (0)	2 (4.4)	1	3 (10.0)	0 (0)	**0.015**
*PDGFRA alteration*	0 (0)	2 (4.8)	1	0 (0)	2 (4.4)	1	10 (33.3)	8 (9.1)	**0.004**
*PDGFRAamp*	0 (0)	1 (2.4)	1	0 (0)	1 (2.2)	1	10 (33.3)	8 (9.1)	**0.004**
*PDGFRAmut*	0 (0)	1 (2.4)	1	0 (0)	2 (4.4)	1	6 (20.0)	3 (3.4)	**0.011**
*PPM1Dmut*	0 (0)	0 (0)	/	2 (18.2)	0 (0)	**0.034**	0 (0)	1 (1.1)	1
*IDH1/2mut*	5 (83.3)	37 (88.1)	0.573	9 (81.8)	31 (67.4)	0.567	12 (40.0)	13 (14.8)	**0.005**
*MGMTmet*	4 (66.7)	27 (64.3)	1	7 (63.7)	32 (69.6)	0.985	14 (46.7)	31 (35.2)	0.284
*TERTmut*	3 (50.0)	23 (54.8)	1	9 (81.8)	21 (45.7)	**0.044**	13 (43.3)	51 (58.0)	0.205
*BRAFmut*	0 (0)	4 (9.5)	1	0 (0)	3 (6.5)	1	0 (0)	2 (2.3)	1
*ATRXmut*	2 (33.3)	9 (21.4)	0.897	1 (9.1)	14 (30.4)	0.288	8 (26.7)	12 (13.6)	0.156
*EGFRamp*	0 (0)	1 (2.4)	1	1 (9.1)	4 (8.7)	1	4 (13.3)	25 (28.4)	0.140

*Bold face type indicates statistical significance.*

**TABLE 2:** Correlative factors of ccGBM analyzed by multivariate logistic regression.

**Variates**	**OR (95%CI)**	***P* value**
Age	0.98 (0.94–1.02)	0.379
Gender		
Female	1	
Male	0.42 (0.16–1.10)	0.076
*MYCN*	2.83 × 10^9^ (0-∞)	1
*PDGFRA*		
Not Alteration	1	
Alteration	4.91 (1.55–15.52)	**0.007**
*IDH1/2*		
Not Alteration	1	
Alteration	2.05 (0.59–7.20)	0.261

*CI, Confidence interval; Bold face type indicates statistical significance.*

### Association of *PDGFRA* Alterations With the Survival Rate of GBM Patients

Because of the small sample size of patients with ccGBM *MYCN* alterations (only three patients), we only analyzed the association between *PDGFRA* alteration and the survival rate of GBM patients. The GBM patients were divided into the ccGBM with *PDGFRA* amplified group (ccGBM with *PDGFRAamp*), the non-ccGBM with no *PDGFRA* amplification group (non-ccGBM with *PDGFRAnon-amp*), the ccGBM with *PDGFRA* mutation combined with amplification group (ccGBM of *PDGFRAamp-mut*), and the non-ccGBM with wild type *PDGFRA* with no amplification (non-ccGBM with *PDGFRA-w, non-amp*) group. To avoid the influence of other factors, these patients were matched according to *IDH 1/2*, *TERT, MGMTmet, and EGFRamp* status. Other clinical characteristics were also matched. The ccGBM with *PDGFRAamp* group had a shorter median PFS (8.6 vs. 13.5 months, *P* = 0.025) and OS (12.4 vs. 17.9 months, *P* = 0.022) rate than the non-ccGBM with *PDGFRAnon-amp* group. The ccGBM with *PDGFRAamp-mut* group had a shorter median PFS (7.6 vs. 8.9 months, *P* = 0.022) and OS (9.6 vs. 17.8 months, *P* = 0.006) rate than the ccGBM with *PDGFRA-w, non-amp* group. The ccGBM with *PDGFRAamp-mut* group also had a lower median EOR value than the non-ccGBM with *PDGFRA-w, non-amp* group (95.97 vs. 100%, *P* = 0.045) (Table 3).

**TABLE 3:** Baseline characteristics and survival of matched ccGBM and non-ccGBM.

**Variables**	**ccGBM of *PDGFRAamp***	**Non-ccGBM of *PDGFRAnon-amp***	***P* value**	**ccGBM of *PDGFRAamp-mut***	**Non-ccGBM of *PDGFRA-w, non-amp***	***P* value**
Total	10	10		6	12	
Age (years)	44.9 ± 14.1	47.5 ± 12.9	0.672	45.3 ± 13.5	48.0 ± 13.5	0.698
Molecular alterations (N [100%])						
*IDH1/2mut*	4 (40.0)	10 (40.0)	1	1 (16.7)	2 (16.7)	1
*MGMTmet*	6 (60.0)	6 (60.0)	1	3 (50.0)	6 (50.0)	1
*TERTmut*	5 (50.0)	4 (40.0)	1	5 (83.3)	8 (66.7)	0.615
*EGFRamp*	2 (20.0)	1 (10.0)	1	1 (16.7)	2 (16.7)	1
Tumor Volume (cm^3^)	54.47 ± 23.25	49.54 ± 42.03	0.749	56.01 ± 17.91	44.17 ± 29.87	0.388
EOR (% [Range])	98.35 (79.33–100)	100 (82.83–100)	0.326	95.97 (87.38–100)	100 (93.28–100)	**0.045**
Radiotherapy (N [%])	10 (100)	9 (90.0)	1	6 (100.0)	11 (91.7)	1
TMZ cycles	5.1 ± 4.1	7.7 ± 4.4	0.189	2.8 ± 3.0	7.3 ± 6.7	0.141
Median PFS (95%CI)	8.6 (7.7–9.5)	13.5 (12.1–14.9)	**0.025**	7.6 (5.3–9.9)	8.9 (8.1–9.7)	**0.022**
Median OS (95%CI)	12.4 (9.9–14.9)	17.9 (16.5–19.3)	**0.022**	9.6 (4.9–14.3)	17.8 (10.0–25.6)	**0.006**

*CI, Confidence interval; Bold face type indicates statistical significance.*

### Association Between *PDGFRA* Alterations and the Survival Rate of ccGBM Patients

**FIGURE 3:**
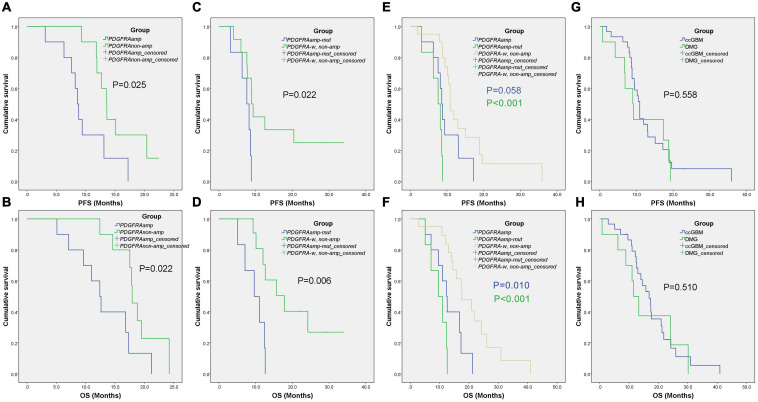
Kaplan-Meier survival curves of different groups. The ccGBM of *PDGFRAamp* vs. non-ccGBM of *PDGFRAnon-amp* **(A,B)**, ccGBM of *PDGFRAamp-mut* vs. non-ccGBM of *PDGFRA-w, non-amp* **(C,D)**, ccGBM of *PDGFRA-w, non-amp* compared to ccGBM of *PDGFRAamp* and *PDGFRAamp-mut* **(E,F)**, and ccGBM vs. DMG **(G,H)**.

To test the influence of *PDGFRA* alterations on the survival rate of ccGBM patients, ccGBM patients were divided into the *PDGFRAamp* group, the *PDGFRAamp-mut* group and the *PDGFRA-w*, and *non-amp* group. The clinical outcomes and survival rates were compared between the groups (Table 4). The ccGBM with *PDGFRAamp* group had a shorter median PFS (8.6 vs. 10.9 months, *P* = 0.058) and OS (12.4 vs. 17.6 months, *P* = 0.010) rate than the *PDGFRA-w, non-amp* group. The ccGBM with *PDGFRAamp-mut* group had a shorter median PFS (7.6 vs. 10.9 months, *P* < 0.001) and OS (9.6 vs. 17.6 months, *P* < 0.001) rate than the *PDGFRA-w, non-amp* group. The survival curves are presented in Figure 3.

**TABLE 4:** Comparison of ccGBM in different PDGFRA alteration status.

**Variables**	**ccGBM *PDGFRA-w, non-amp***	**ccGBM *PDGFRAamp***	*P* value*	**ccGBM *PDGFRAamp-mut***	*P* value*
Total	20	10		6	
Age (years)	49.9 ± 12.1	44.9 ± 14.1	0.321	45.3 ± 13.5	
Gender (N [%])			0.705		0.365
Male	8 (40.0)	5 (50.0)		4 (66.7)	
Female	12 (60.0)	5 (50.0)		2 (33.3)	
Molecular alterations (N [100%])					
*IDH1/2mut*	7 (35.0)	5 (50.0)	0.461	1 (16.7)	0.628
*MGMTmet*	8 (40.0)	6 (60.0)	0.701	3 (50.0)	1
*TERTmut*	8 (40.0)	5 (50.0)	0.705	5 (83.3)	0.160
*EGFRamp*	3 (15.0)	1 (10.0)	1	1 (16.7)	1
Tumor Volume (cm^3^)	51.85 ± 29.79	54.47 ± 23.25	0.810	56.01 ± 17.91	0.394
EOR (% [Range])	100 (88.77–100)	98.35 (79.33–100)	0.205	95.38 (87.38–100)	0.078
Radiotherapy (N [%])	16 (80.0)	10 (100)	0.272	6 (100.0)	0.542
TMZ cycles (N [IQR])	5 (0.5–6)	3.5 (1.50–8.25)	0.929	2.5 (0–5)	0.265
Median PFS (95%CI)	10.9 (10.0–11.8)	8.6 (7.7–9.5)	**0.058**	7.6 (5.3–9.9)	**<0.001**
Median OS (95%CI)	17.6 (11.5–23.7)	12.4 (9.9–14.9)	**0.010**	9.6 (4.9–14.3)	**<0.001**

*compared with the ccGBM of *PDGFRA non-amp* and wild type; CI, Confidence interval; IQR, interquartile range; Bold face type indicates statistical significance.

### Influence of *PDGFRA* Alterations on ccGBM Prognosis

Multivariate analysis showed that *PDGFRAamp*, the EOR value and number of TMZ cycles correlated with the OS rate of ccGBM patients (*P* < 0.05). *PDGFRAamp* caused a shorter OS rate (HR = 5.07 [95% CI: 1.52–16.89], *P* = 0.008). *PDGFRAamp* was also significantly associated with the PFS rate of ccGBM patients (HR = 3.08 [95% CI: 1.02–9.35], *P* = 0.047). *PDGFRAamp-mut* also had a significant negative influence on the PFS rate (HR = 13.16 [95% CI: 3.19–54.40], *P* < 0.001) and OS rate (HR = 16.36 [95% CI: 2.66–100.70], *P* = 0.003) of ccGBM patients in the multivariate Cox regression analysis (Tables 5, 6).

**TABLE 5:** Influence of *PDGFRAamp* on prognosis of ccGBM by multivariate cox regression.

**Multivariate Analysis**	***PDGFRA amp***	**Tumor volume**	**EOR**	**Radiotherapy**	**TMZ Cycles**
HR for PFS (95% CI)	3.08 (1.02–9.35)	1.01 (0.98–1.03)	0.98 (0.90–1.03)	0.31 (0.05–2.01)	0.90 (0.76–1.08)
*P* value	**0.047**	0.602	0.609	0.221	0.258
HR for OS (95% CI)	5.07 (1.52–16.89)	1.02 (1.00–1.04)	0.90 (0.82–1.00)	0.42 (0.06–2.69)	0.79 (0.65–0.95)
*P* value	**0.008**	0.086	**0.043**	0.357	**0.014**

*Bold face type indicates statistical significance.*

**TABLE 6:** Influence of *PDGFRAamp-mut* on prognosis of ccGBM by multivariate cox regression.

**Multivariate Analysis**	***PDGFRAamp-mut***	**Tumor volume**	**EOR**	**Radiotherapy**	**TMZ Cycles**
HR for PFS (95% CI)	13.16 (3.19–54.40)	1.00 (0.98–1.02)	0.95 (0.87–1.03)	0.23 (0.03–1.59)	0.98 (0.82–1.17)
*P* value	**<0.001**	0.788	0.240	0.136	0.821
HR for OS (95% CI)	16.36 (2.66–100.70)	1.01 (1.00–1.04)	0.84 (0.75–0.95)	0.24 (0.03–1.89)	0.88 (0.73–1.05)
*P* value	**0.003**	0.244	**0.004**	0.177	0.162

*Bold face type indicates statistical significance.*

### Comparison of ccGBM With Diffuse Midline Glioma

Molecular alterations were compared between ccGBM and diffuse midline glioma (DMG). The results demonstrated that the incidences of *PDGFRA* alterations were not significantly different (33.3 vs. 70.0%, *P* = 0.097) between the two groups. The incidences of *PDGFRAamp* or *mut* (PDGFRAamp/mut) were also similar between groups (*P* > 0.05). The *TERT* mutation incidence of ccGBM was higher than that of DMG, while the *ATRX* mutation incidence of ccGBM was lower than that of DMG. The median PFS and OS rates were not significantly different between the two groups (Table 7 and Figure 3).

**TABLE 7:** Comparison of molecular alterations and survival between ccGBM and diffuse midline glioma.

**Variables**	**ccGBM**	**Diffuse midline glioma**	***P* value**
Total	30	10	
Age (years)	48.2 ± 12.8	33.2 ± 11.8	**0.002**
Gender (N [%])			0.705
Male	13 (43.3)	5 (50.0)	1
Female	17 (56.7)	5 (50.0)	
Molecular alterations (N [100%])			
*IDH1/2mut*	12 (40.0)	1 (10.0)	0.172
*MGMTmet*	14 (46.7)	3 (30.0)	0.580
*TERTmut*	13 (43.3)	0 (0)	**0.032**
*ATRXmut*	8 (26.7)	7 (70.0)	**0.038**
*EGFRamp*	4 (13.3)	0 (0)	0.543
*PDGFRA* alteration	10 (33.3)	7 (70.0)	0.097
*PDGFRAamp*	10 (33.3)	2 (20.0)	0.693
*PDGFRAmut*	6 (20.0)	5 (50.0)	0.152
Tumor Volume (cm^3^)	52.72 ± 27.40	41.14 ± 34.28	0.179
EOR (% [Range])	100 (79.33–100)	86.94 (10.97–100)	0.118
Radiotherapy (N [%])	25 (83.3)	9 (90.0)	1
TMZ cycles (N [IQR])	5 (1.5–6)	5.5 (2.25–8.25)	0.600
Median PFS (95%CI)	10.9 (10.0–11.8)	9.0 (5.4–12.6)	0.558
Median OS (95%CI)	16.8 (12.6–21.0)	11.5 (8.3–14.7)	0.510

*CI, Confidence interval; IQR, interquartile range; Bold face type indicates statistical significance.*

## Discussion

Platelet-derived growth factor receptor α (PDGFRα) is a transmembrane receptor encoded by *PDGFRA*. It has 5 immunoglobulin-like repeats in its extracellular domain and a tyrosine kinase (TK) in its intracellular domain. The activation of PDGFRα by its ligand can induce multiple downstream signal transduction pathways that promote oncogenesis, including the PI3K/AKT, RAS/MAP kinase, Src kinase family, JAK/STAT, and PLC/PKC pathways (Blume-Jensen and Hunter, 2001; Verhaak et al., 2010). PDGFRA is the second most frequently mutated receptor tyrosine kinase (RTK) in GBM, followed by EGFR (Verhaak et al., 2010). Several *PDGFRA* alterations have been detected in gliomas, including amplification, missense mutation, frameshift mutation, in-frame insertion or deletion, and rearrangement (Martinho et al., 2009; Ozawa et al., 2010). Some previous studies have demonstrated a high incidence of *PDGFRAamp* in GBM of adults, ranging from 7.4 to 29.8% (Joensuu et al., 2005; Cancer Genome Atlas Research Network, 2008; Martinho et al., 2009; Ozawa et al., 2010; Verhaak et al., 2010; Nobusawa et al., 2011; Alentorn et al., 2012; Kessler et al., 2018; Gonzalez-Tablas et al., 2020; Shen et al., 2020). Pediatric (<18 years old) GBM had the higher incidence of *PDGFRAamp* which ranged from 14.4 to 38.6% (Paugh et al., 2010; Phillips et al., 2013; Johnson et al., 2017; Korshunov et al., 2017). The incidence of *PDGFRAmut* in GBM of adults ranged from 0 to 5.2% (Cancer Genome Atlas Research Network, 2008; Verhaak et al., 2010; Alentorn et al., 2012; Shen et al., 2020). The pediatric GBM also had the higher incidence of *PDGFRAmut* in a previous study (11.1%) (Johnson et al., 2017). Neither *PDGFRAamp* nor *PDGFRAmut* were found to be associated with the survival rate of adult GBM patients in previous studies. Only Puputti et al. (2006) and Alentorn et al. (2012) reported the negative influence of *PDGFRAamp* on the survival rate of glioma patients with WHO grade II and III tumors (Table 8). Some previous studies demonstrated the higher incidence of *PDGFRA* alterations of pediatric GBM compared to adult GBM. The negative influence of *PDGFRAamp* on survival of pediatric GBM was also reported in a previous study (Korshunov et al., 2017). We only included adult patients with GBM in our cohort and found the incidences of *PDGFRAamp/mut* were similar with those of previous studies (15.3 and 7.6%, respectively). While the ccGBM in our cohort had the higher incidences of *PDGFRAamp/mut* (33.3 and 20.0%, respectively) which were similar with those of pediatric GBM. Because the previous studies of pediatric GBM also included many infra-tentorial tumors that invaded brain stem or cerebellum, we inferred that higher incidences of *PDGFRA* alterations were not only associated with the younger age of patients, but also the midline structures invasion (such as corpus callosum, thalamus and brainstem) of tumor.

**TABLE 8:** Summary of studies on patients with GBM of *PDGFRA* alterations.

***PDGFRA* alterations**	**Related references**	**Patients**	**N (incidence)**	**Influence on overall survival**
Missense mutation	Joensuu et al. (2005)	Adults	47 (0%)	NR
	Cancer Genome Atlas Research Network (2008)	Adults	91 (1.1%)	NR
	Verhaak et al. (2010)	NR	116 (3.4%)	NR
	Alentorn et al. (2012)	Adults	284 (1.4%)	NR
	Shen et al. (2020)	Adults	58 (5.2%), GBM = 36	NR
	Johnson et al. (2017)	Pediatrics	90 (11.1%)	NR
	Present study	Adults	118 (7.6%)	Negative
		ccGBM	30 (20.0%)	Negative
Amplification	Joensuu et al. (2005)	Adults	47 (29.8%)	No association
	Cancer Genome Atlas Research Network (2008)	Adults	206 (11.2%)	NR
	Martinho et al. (2009)	NR	19 (15.8%)	No association
	Verhaak et al. (2010)	Adults	170 (25.9%)	NR
	Ozawa et al. (2010)	NR	63 (23.8%)	NR
	Nobusawa et al. (2011)	NR	390 (8.5%)	No association
	Alentorn et al. (2012)	Adults	284 (9.2%)	No association
	Kessler et al. (2018)	NR	85 (12.9%)	NR
	Gonzalez-Tablas et al. (2020)	Adults	68 (7.4%)	No association
	Shen et al. (2020)	Adults	58 (5.2%), GBM = 36	NR
	Paugh et al. (2010)	Pediatrics = 58	58 (17.0%)	NR
		Adults = 189	189 (11.0%)	NR
	Phillips et al. (2013)	Pediatric = 57	57 (38.6%)	No association
		Adults = 160	160 (22.5%)	No association
	Korshunov et al. (2017)	Pediatrics	87 (16.1%)	Negative
	Johnson et al. (2017)	Pediatrics	90 (14.4%)	NR
	Present study	Adults	118 (15.3%)	Negative
		ccGBM	30 (33.3%)	Negative
Rearrangement	Ozawa et al. (2010)		212 (2.8%)	NR
	Alentorn et al. (2012)	Adults	284 (0.4%)	NR
	Present study	Adults	118 (0%)	NR
In-frame deletion	Shen et al. (2020)	Adults	58 (1.7%)	NR
	Present study	Adults	118 (0%)	NR

*N, total patients; NR, not reported.*

Diffuse midline glioma invading midline structures (thalamus, pons, and medulla oblongata, etc.) was identified to have a much higher incidence of *PDGFRAamp/mut* in previous studies [30.2% in Paugh et al. (2011), 21.3% in Castel et al. (2015) and 33.3% in Paugh et al. (2011) and Dono et al. (2020)]. As the largest interhemispheric fiber bundle, the corpus callosum is another midline structure that is often invaded by glioma. ccGlioma was also considered to have a higher incidence of *PDGFRA* alterations than non-ccGlioma in a previous study (Shen et al., 2020). Furthermore, ccGlioma was indicated to have a poorer prognosis than non-ccGlioma, and ccGBM had an even shorter median OS, ranging from 7.0 to 15.0 months after surgery (Dziurzynski et al., 2012; Chaichana et al., 2014; Chen et al., 2015; Burks et al., 2017; Dayani et al., 2018; Opoku-Darko et al., 2018; Forster et al., 2020; Franco et al., 2020). Therefore, we hypothesized that *PDGFRA* alterations may play an important role in GBM and lead to a higher incidence of CC invasion and a poorer prognosis.

In our analysis of our study data, ccGBM indeed had a higher incidence of *PDGFRA* alterations than the non-ccGBM. Shen et al. (2020) only demonstrated the higher incidence of PDGFRA alterations in ccGlioma. We found that most *PDGFRA* alterations occurred in ccGBM, and no *PDGFRA* alterations were found in ccGlioma classified as WHO grade II and III. Thus, ccGBM, but not grade II and III ccGliomas, can be characterized by *PDGFRA* alterations (OR = 4.97, *P* = 0.007). Furthermore, among the *PDGFRA* alterations, ten patients had *PDGFRAamp*, and six patients had *PDGFRA* missense mutations. Frameshift mutations, in-frame insertions or deletions, and rearrangements were not identified in our study. Although these types of alterations were reported in some previous studies, their incidences were very low in GBM (Ozawa et al., 2010; Shen et al., 2020). Only *PDGFRA* amplification and missense mutation were thought to drive CC invasion in GBM in our study. Interestingly, *PDGFRAamp* accounted for 100% of *PDGFRA* alterations in ccGBM or non-ccGBM, which may indicate the more important initial effect of *PDGFRAamp* in causing CC invasion. Previous studies did not identify a correlation between *PDGFRA* alterations and the survival rate of GBM patients. Our analysis showed that the ccGBM with *PDGFRAamp* group had shorter median PFS and OS rate than the non-ccGBM with *PDGFRAnon-amp* group. The ccGBM with *PDGFRAamp-mut* group had a shorter median PFS and OS rate than the non-ccGBM of *PDGFRA-w, non-amp* group. The ccGBM with *PDGFRAamp-mut* group also had lower EOR values. These results demonstrated that *PDGFRA* alterations, especially *PDGFRAamp* combined with *PDGFRAmut*, can cause poorer prognosis in GBM patients by increasing the invasive ability of tumors (invading CC) and increasing the rate of incomplete resection or residual tumors. The ccGBM with *PDGFRAamp or PDGFRAamp-mut* group also had a significantly shorter PFS and OS rate than the ccGBM with *PDGFRA-w, non-amp* group, which highlighted the obvious negative influence of *PDGFRA* alterations on the survival rate of ccGBM patients.

Compared to the 10 patients with DMG, although the ccGBM had no statistical differences of *PDGFRAamp/mut* and total *PDGFRA* alteration incidences, because of the limited samples of DMG, we cannot conclude that they both had similar molecular characteristics of *PDGFRA*. But we can infer that the ccGBM group had a characteristic change in *PDGFRA*, which can predict the invasion of midline structures (such as the CC) and a poorer prognosis than that associated with common GBM. Castel et al. (2015) introduced a subtype of DMG with a K27M mutation in H3.3 (*H3F3A*) that had a prometastatic gene expression signature with *PDGFRA* activation and was also located in midline regions other than the pons. *PDGFRAamp/mut* was seen in this subtype of DMG but not in the H3.1 subtype with a *HIST1H3B/C* mutation. Along with *PDGFRAamp/mut*, H3.3 was a more common subgroup of DMG and had a poorer prognosis than H3.1 (Castel et al., 2015). In our series of DMG patients, all ten had H3.3 *H3F3A* mutations and had a high incidence of *PDGFRAamp/mut*. ccGBM had a similar incidence of *PDGFRAamp/mut* and survival rate to DMG. Therefore, it can be hypothesized that ccGBM with *PDGFRAamp/mut* is a subtype of GBM that has a poor prognosis similar to DMG with H3.3 mutations. Although *PDGFRAamp* and *PDGFRAamp-mut* both influenced the PFS and OS rate of ccGBM patients in the multivariate prognostic analysis, a higher EOR value and more TMZ cycles is indicative of an improved OS time in these patients. Radiotherapy was also found to improve the PFS (HR = 0.20 [95% CI: 0.07–0.60, *P* = 0.003]) and OS (HR = 0.15 [95% CI: 0.05–0.47], *P* = 0.001) rates of ccGBM patients in the univariate analysis but was not found to be associated with survival in the multivariate analysis. We hypothesize that this is because only five patients did not receive postoperative radiotherapy, which may have led to the underestimation of the effect of radiotherapy. Therefore, it is still suggested to perform aggressive resection and postoperative radiochemotherapy for patients with ccGBM with *PDGFRAamp/mut*.

*PDGFRAamp/mut* can cause overexpression of PDGFRα, thus promoting the proliferation and survival of tumor cells through the multiple signaling pathways mentioned above. PDGFR inhibitors are a promising drug to treat glioma. Several antitumor agents targeting PDGFR have been developed, such as imatinib, sorafenib, and nilotinib. These inhibitors have been shown to successfully inhibit GBM cells *in vitro* and in animal experiments (Ren et al., 2009; Ranza et al., 2010). Some clinical trials failed to prove the antitumor effects of PDGFR inhibitors in GBM, which may be due to the emergence of resistance (De Witt Hamer, 2010; Song et al., 2018). Because ccGBM was identified to have a higher incidence of *PDGFRAamp/mut* than non-ccGBM in this study, PDGFR inhibitors may be effective for patients with this specific type of GBM. Further clinical trials are needed to explore the effect of PDGFR inhibitors on ccGBM, especially ccGBM with *PDGFRAamp/mut*.

Some limitations existed in this study. First, not all patients underwent molecular tests, which may lead to selection bias. Second, the incidences of *MYCN* alterations were significantly different between ccGBM and non-ccGBM. However, because of the small sample size of patients with these alterations (only three patients), the association between *MYCN* alteration and the occurrence of ccGBM and patient survival rates could not be analyzed. Third, the limited samples of DMG made it difficult to conclude the similarity of molecular and survival between DMG and ccGBM. Last, further analysis of TCGA data for molecular alterations in ccGBM were needed to validate the findings of this study in our future work.

## Conclusion

Platelet derived growth factor receptor alpha alterations are associated with the occurrence of ccGBM. The ccGBM with *PDGFRAamp/mut* group had a poorer prognosis than the non-ccGBM and ccGBM with *PDGFRA-w, non-amp* groups. ccGBM may have a similar incidence of *PDGFRAamp/mut* to DMG with an H3F3A mutation, and a similar survival rate. ccGBM with *PDGFRAamp/mut* can be classified as a single subtype of GBM that still benefits from gross total resection and radiochemotherapy. PDGFR inhibitors may be a promising treatment method for this type of GBM. Large-sample studies are needed to clarify the molecular mechanisms of ccGMB and potential treatment strategies.

## Data Availability Statement

The original contributions presented in the study are publicly available. This data can be found here: https://datadryad.org/stash/landing/show?id=doi%3A10.5061%2Fdryad.7d7wm37v8.

## Ethics Statement

The studies involving human participants were reviewed and approved by Ethics committee of Chinese PLA General Hospital. The patients/participants provided their written informed consent to participate in this study.

## Author Contributions

MC: conceptualization, methodology, software, formal analysis, resources, data curation, investigation, writing-original draft, writing-reviewing and editing, and visualization. XG: data curation, investigation, and writing-reviewing and editing. YC: software, data curation, visualization, and writing-reviewing and editing. MZ: software, validation, data curation, and investigation. HL and HC: data curation and investigation. CS: resources, supervision, and writing-reviewing and editing. XM: conceptualization, methodology, validation, resources, supervision, project administration, and writing-reviewing and editing. All authors contributed to the article and approved the submitted version.

## Conflict of Interest

YC was employed by the company Xian Janssen Pharmaceutical Ltd. The remaining authors declare that the research was conducted in the absence of any commercial or financial relationships that could be construed as a potential conflict of interest.

## Publisher’s Note

All claims expressed in this article are solely those of the authors and do not necessarily represent those of their affiliated organizations, or those of the publisher, the editors and the reviewers. Any product that may be evaluated in this article, or claim that may be made by its manufacturer, is not guaranteed or endorsed by the publisher.

## Abbreviations

*ATRX*: ATRX chromatin remodeler
*BCOR*: *BCL6* corepressor
*BRAF*: B-Raf proto-oncogene, serine/threonine kinase
CC: corpus callosum
DMG: diffuse midline glioma
DNA: deoxyribonucleic acid
*EGFR*: epidermal growth factor receptor
EOR: extent of resection
GBM: glioblastoma multiforme
*H3F3A*: H3 histone, family 3A
*HIST1H3B/C*: histone cluster 1, H3B/C
HR: hazard ratio
IDH: isocitrate dehydrogenase
*MGMT*: O-6-methylguanine-DNA methyltransferase
*MYCN*: *MYCN* proto-oncogene, bHLH transcription factor
NGS: next-generation DNA sequencing
OR: odds ratio
OS: overall survival
*PDGFRA*: platelet derived growth factor receptor alpha
PFS: progression-free survival
*PPM1D*: protein phosphatase, Mg2+/Mn2+ dependent 1D
RTK: receptor tyrosine kinase
*TERT*: telomerase reverse transcriptase
TMZ: temozolomide
WHO: World Health Organization.
